# A novel process of viral vector barcoding and library preparation enables high-diversity library generation and recombination-free paired-end sequencing

**DOI:** 10.1038/srep37563

**Published:** 2016-11-22

**Authors:** Marcus Davidsson, Paula Diaz-Fernandez, Oliver D. Schwich, Marcos Torroba, Gang Wang, Tomas Björklund

**Affiliations:** 1 Molecular Neuromodulation, Department of Experimental Medical Science, Lund University, 221 84 Lund, Sweden

## Abstract

Detailed characterization and mapping of oligonucleotide function *in vivo* is generally a very time consuming effort that only allows for hypothesis driven subsampling of the full sequence to be analysed. Recent advances in deep sequencing together with highly efficient parallel oligonucleotide synthesis and cloning techniques have, however, opened up for entirely new ways to map genetic function *in vivo*. Here we present a novel, optimized protocol for the generation of universally applicable, barcode labelled, plasmid libraries. The libraries are designed to enable the production of viral vector preparations assessing coding or non-coding RNA function *in vivo*. When generating high diversity libraries, it is a challenge to achieve efficient cloning, unambiguous barcoding and detailed characterization using low-cost sequencing technologies. With the presented protocol, diversity of above 3 million uniquely barcoded adeno-associated viral (AAV) plasmids can be achieved in a single reaction through a process achievable in any molecular biology laboratory. This approach opens up for a multitude of *in vivo* assessments from the evaluation of enhancer and promoter regions to the optimization of genome editing. The generated plasmid libraries are also useful for validation of sequencing clustering algorithms and we here validate the newly presented message passing clustering process named Starcode.

Massively parallel *in vitro* or *in vivo* interrogation of genetic sequence or protein function has recently seen a rapid development. This is due to a combination of technological developments such as gene chip array oligonucleotide synthesis^1^, highly efficient and flexible cloning methods^2^, efficient viral vector production techniques^3^ and not least the extraordinary reduction in next-generation sequencing (NGS) costs. Broad diversity plasmid libraries have been successfully applied in almost every area of biology from virology^4^ to hematopoiesis^5^,^6^. To successfully evaluate and characterize the library and functional outcome in a clean, unbiased and systematic way is quintessential for this category of studies. In general, the experimental design can be divided into four components (i) Design of the expression system, *i.e.,* the plasmid where the functional sequences are studied, *e.g.,* a cis-regulating promoter or enhancer fragment is driving the expression of a fluorescent protein^7^,^8^,^9^. (ii) Generation of a suitable library of genetic sequences to insert into the expression system, *e.g.,* fragmentation of genomic DNA^7^ or synthesis of oligonucleotides using gene chip arrays^9^. (iii) Efficient insertion of the fragment library into the expression system, *e.g.,* using dA/dT ligation^10^, Gateway cloning^11^ or Gibson assembly^2^. (iv) Once a plasmid library of sufficient diversity is generated, the biological function can be evaluated for each sequence, either independently or multiplexed in the same sample.

While undoubtedly successful in a number of studies, this approach puts a high demand on the methods utilized to ensure that the resulting plasmid library displays a uniform and predictable distribution of the studied sequences and that their exact identity can be reproducibly determined. As exemplified above, one useful application of this approach is to systematically map the cis-regulating elements (CRE) of tissue specific promoter regions through the insertion of genomic sequence fragment library into a recombinant AAV vector^7^. However, in this case, and in a number of other potential applications, the efficiency readout is carried out through the NGS evaluation of expressed RNA from the AAV vector and the CRE is not itself expressed in the mRNA. Thus, an unique identifier (a.k.a. barcode) needs to be unambiguously linked to the corresponding CRE and be expressed in the mRNA. The link between barcode and assessed genomic fragment is usually made through sequencing of the plasmid library before the production of the viral vector. This strategy depends on sequencing of the barcode at the same time as the fragment which either means using a long sequencing technology such the Pacific Biosciences RSII sequencer (PacBio) or using paired-end sequencing in the Illumina sequencers where one read covers the barcode and the other the fragment. Once this sequencing has generated a complete look-up table, then a short read single-end sequencing is all that is required *in vivo*, counting the barcodes, to determine the functional contribution of each fragment.

One great advantage with the PacBio sequencing is that it is a single molecule sequencing technology and is the only one to date that can deliver sequencing results that are entirely free from polymerase based pre-amplification (*e.g.,* by PCR for NEXTERA index addition, emRCR for Ion Torrent, and PCR for cluster generation in the Illumina flow cell). Unfortunately, the cost per read is multiple orders of magnitude higher than that for the Illumina technology and thus, it is not very suitable for applications with high diversity libraries (greater than one million unique barcodes).

PCR in a solution is hampered by a number of biophysical properties that can severely disrupt and/or induce bias in the sequencing of genetic fragments combined with molecular barcodes. For example, as most designs include a static stretch of nucleotides between the genetic fragment and the barcode (incorporating the functional domains such as a minimal promoter and fluorescent protein in the case of the CRE studies described above) which can be multiple hundreds of base pair long, the risk of PCR template switching is very high. Template switching in this region is highly deleterious as this would link the genetic fragment to the false barcode^9^,^12^,^13^,^14^.

In this paper we present the development and characterization of a novel design protocol that enables efficient DNA fragmentation, cloning, barcode labelling and lookup table generation using cheap and simple reagents that enable the generation of libraries with multiple million unique barcodes. Furthermore, this method is designed to allow for recombination-free sequencing using PCR based paired-end sequencing. We utilize this platform to validate the novel algorithm of Starcode^15^ and show that this method, if used restrictively can outperform other reduction techniques without falsely clustering barcodes.

## Results

### Creation and validation of barcoded DNA plasmid libraries using dUTP-PCR and DNA fragmentation

**Figure 1:**
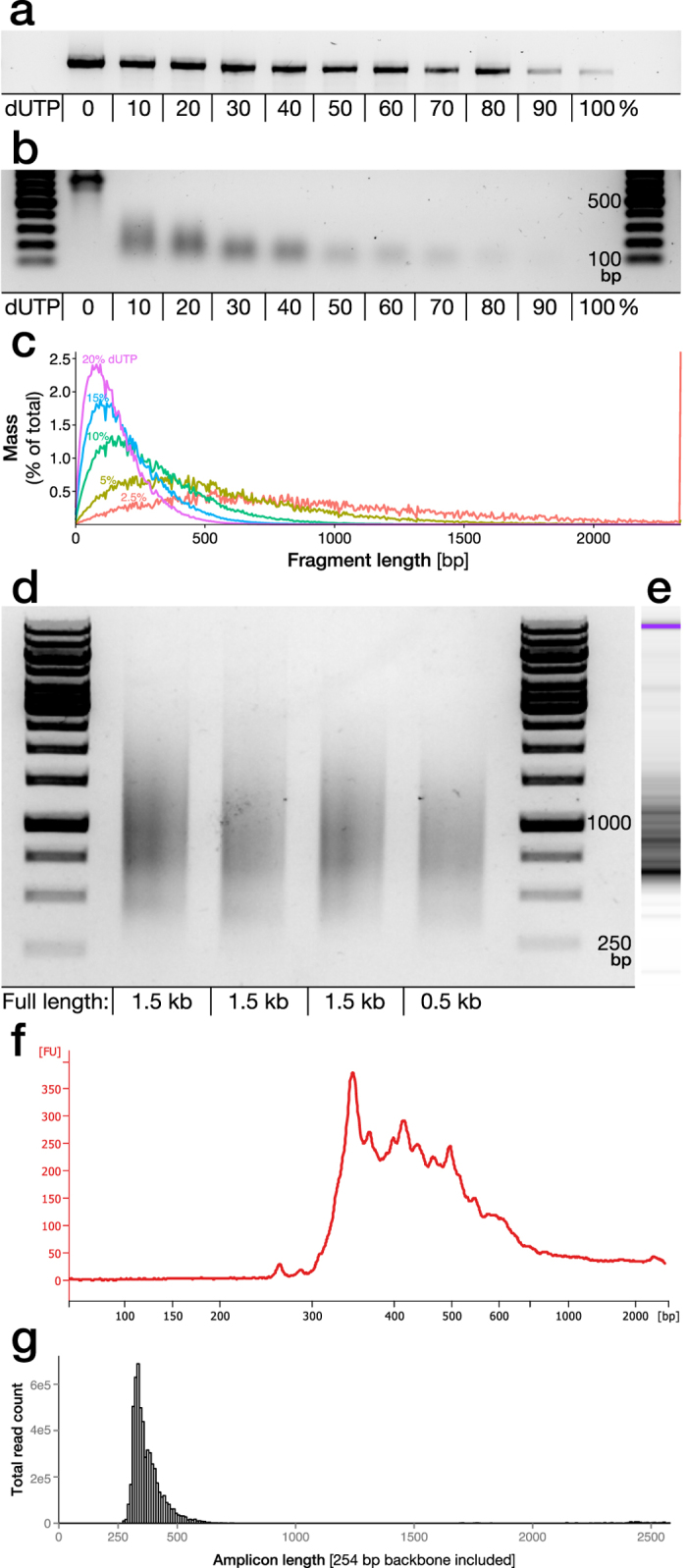
Fragmentation of genetic sequences for insertion in barcode labelled plasmids. **(a)** A novel version of the Pfu-based proof-reading polymerase “Phusion U” allows for insertion of dUTP during PCR amplification. However, efficiency of amplification is reduced with increasing dUTP/dTTP fraction as exemplified through amplification of a genomic sequence (1.1 kb long) utilized in library 1. **(b)** The percentage dUTP used in the PCR reaction is determining the distribution of fragment sizes after nicking by Uracil-DNA-Glycosylase (UDG) and NaOH single strand breakage. **(c)** As the cleavage sites are strictly sequence dependent and statistically predictable based on dUTP fraction and insertion frequency it can be simulated using the NExTProg 1.0 software. Here this was modelled using the sequence for library 1. **(d)** The dUTP/UDG fragmentation protocol was applied to three amplicons with different sequences but similar length and GC content (49–52%), aimed for inclusion in library 3, and one shorter amplicon, with 10% dUTP. **(e,f)** The fragments from the first lane were further analysed by higher accuracy electrophoretic analysis (Bioanalyzer) and **(g)** Illumina sequencing was performed after insertion into a plasmid backbone (library 3).

In total, four barcoded DNA plasmid libraries were generated in this project (see Table 1). While the libraries differ in the design to adhere to the optimizations and novel approaches presented in this paper, the one thing they have in common is that they all are designed to utilize genomic fragments from a library to study the function of non-coding RNA. To generate the large diversity of DNA fragments from the same genomic template sequence, DNA was either isolated and amplified from a genomic source (library 1–3) or from plasmid-DNA (library 4) using PCR. Each full length amplicon was then subjected to a PCR amplification with varying concentration of dUTP using either a Taq- or Pfu- polymerase capable of incorporating dUTPs (*e.g.,* Phusion U Hot Start). The Pfu-based proof reading polymerase handles dUTP integration but at a lower efficiency than dTTP (Fig. 1a). The addition of a small fraction Uracil nucleotides in the amplicons allow for unbiased fragmentation of the genomic sequences that is not dependent on either physical shearing or restriction enzyme recognition sites.

Fragmentation of amplified dUTP containing amplicons (library 1) was carried out by Uracil-DNA-Glycosylase (UDG) and NaOH resulting in a fragment size gradient depending on percentage dUTP used in the PCR reaction (Fig. 1b). The cleavage sites are statistically predictable based on the reference sequence where size distribution can be modelled using the available software tool NExTProg 1.0^16^ (Fig. 1c).

An even length distribution, complete sequence coverage and unbiased fragmentation sites are key components for the generation of a complete screening library. To validate the robustness and sequence dependency of the dUTP/UDG fragmentation protocol, we applied it to three amplicons of around 1.5 kb in size (library 3) with a GC content of 49–52% and one shorter (0.5 kb) amplicon with 40% GC content. Based on the *in silico* modelling, a dUTP percentage of 10% was chosen for the dUTP PCR. The fragment distribution from the three long amplicons was highly similar despite differences in the underlying sequence and the shorter amplicon retained the short fragment distribution but naturally lacked the long sequences (Fig. 1d). The fragments from the first lane in Fig. 1d were inserted into a plasmid backbone (library 3) and assessed further using Bioanalyzer technology (Fig. 1e,f) and Illumina sequencing (Fig. 1g). These data confirm that the size distribution is well maintained throughout the cloning process.

### Cloning and barcoding of unique DNA fragments

Deep and detailed dissection of functional sub-domains in large fragment libraries depends on a very high efficiency cloning process. The first limiting step in this process is the ligation of one unique fragment into each plasmid backbone. This should allow large libraries to be generated with relative ease and should not result in the concurrent generation of empty backbones (*i.e.,* missing a genomic fragment). In this study we validated a number of available technologies (Table 2) and found one approach superior to the others.

**Figure 2:**
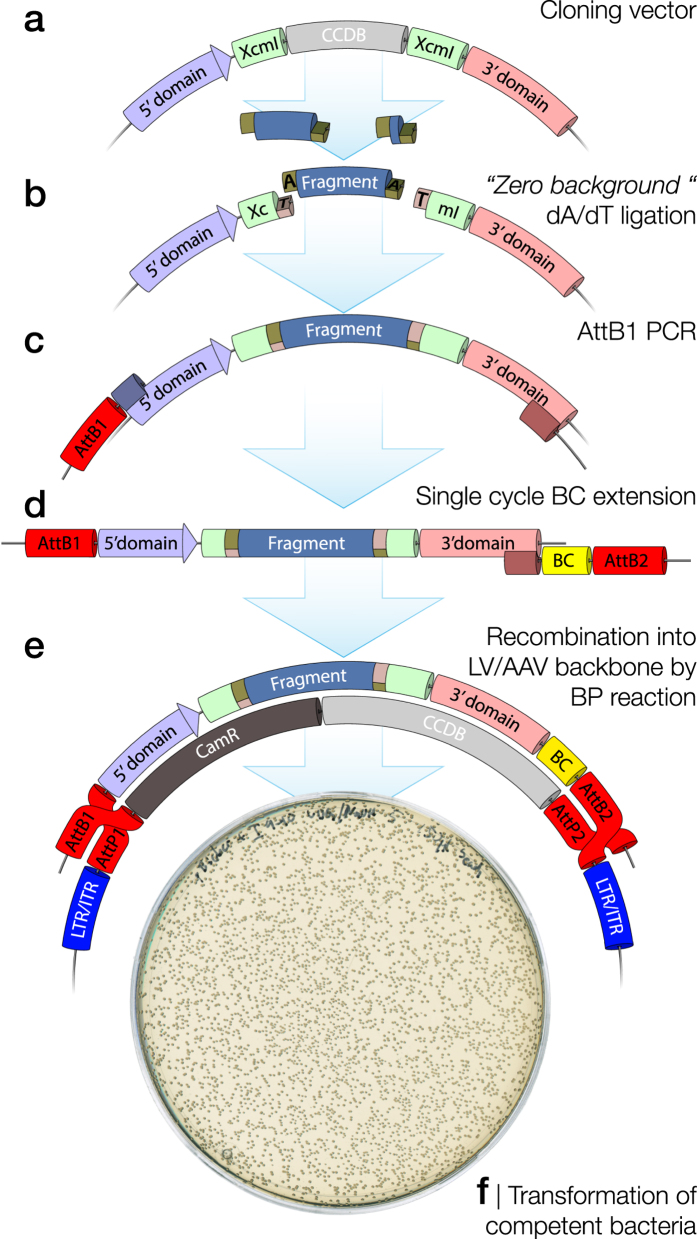
Process for efficient, zero-background, cloning of uniquely barcoded dA-tailed fragment libraries. **(a)** To achieve ligation of one unique fragment into each plasmid backbone, a relatively small cloning vector (5.7 kb) was generated containing a ccdB toxin gene flanked by two specifically tailored XcmI restriction enzyme cleavage sites. **(b)** The sequence is tailored so that XcmI enzyme digestion leaves a single 5′ dT-overhang on both open ends of the backbone generating a T-vector where the dA-tailed DNA fragments can be efficiently ligated without directional bias. **(c)** The cloning vector can have a flexible design with any 5′ and 3′ domain modulated by the inserted fragment. To allow for unique barcoding of each fragment together with the surrounding 5′ and 3′ domains, the region of interest in the cloning vector is then exposed to a two-step PCR amplification where a 5′ AttB1 site is inserted in the first amplification (20 cycles amplification). **(d)** The barcode together with the AttB2 site are added through a PCR with only a single elongation step, ensuring that each unique barcode is utilized only once and is not transferred to other amplicons due to PCR template switching. **(e)** The library of uniquely barcoded PCR amplicons is then inserted into the viral vector backbone using the “Gateway” BP clonase recombination reaction. **(f)** Chemically or electro-competent bacteria are then transformed and a small fraction of the transformation reaction plated for a rough estimation of total number of colonies. Generation of empty backbones (*i.e.,* missing a genomic fragment) is kept to an absolute minimal as any such plasmid would contain the ccdB toxin gene providing negative selection pressure.

A relatively small cloning vector (5.7 kb) was generated containing a ccdB toxin gene flanked by two specifically tailored XcmI restriction enzyme cleavage sites (Fig. 2a). This vector is designed so that bacteria taking up vectors still containing ccdB will be selected against, reducing the risk of empty backbones in the library. The XcmI enzyme leaves a single base overhang that can be tailored to be a 5′ T-overhang on both open ends of the backbone^17^. This results in a T-tailed vector after XcmI digestion and the approach is named by the inventor the “zero background” cloning. DNA fragments generated in Fig. 1c were first converted into blunt oligonucleotides using an end-repaired reaction and then dA-tailed using Klenow fragment polymerase. Finally, the A-tailed fragments were ligated into the T-tailed vectors (Fig. 2b).

The use of a small cloning vector in the step of dA/dT-ligation ensures an efficient reaction resulting in high fragment diversity. However, the final goal of most libraries is to transduce cells *in vitro* or *in vivo* using either lentiviral (LV) or adeno-associated viral (AAV) vector technologies, both requiring large and complex expression plasmids. The library is therefore, in a second step, migrated to an AAV or LV expression vector. This transfer, here mediated by the “Gateway” BP clonase recombination reaction, also allows for a 20 nucleotide degenerate sequence (a molecular barcode) to be inserted into the final construct, positioned at a significant distance from the assessed genetic fragment. The amplification is here conducted in a two-step reaction to add the BP-clonase directional recombination sites (AttB1 & AttB2). In the first step, the AttB1 site was added in a normal PCR reaction together with a reverse primer annealing in the 3′ domain (Fig. 2c). Following this, the AttB2 site was added in a single cycle PCR reaction (Fig. 2d) through a second reverse primer, again binding to the 3′ domain. This primer contains both the genetic barcode and the AttB2 site. This ensures that each unique barcode is utilized only once and is not transferred to other amplicons due to PCR template switching. The library of uniquely barcoded PCR amplicons was subsequently inserted into the viral vector backbone using the BP reaction (Fig. 2e). Chemically or electro-competent bacteria were then transformed and a small fraction of the transformation reaction was plated for a rough estimation of total number of clones (Fig. 2f).

### Development of suitable plasmid backbones for sequencing of barcoded fragment libraries

In this project we have utilized three alternative sequencing paradigms to map fragments to genomic sequences. The sequencing data is also utilized to determine the linkage between barcode and fragment in each plasmid. Deep sequencing of barcodes together with long DNA fragments (up to 2.3 kb in this experiment) combines two challenges; high sequencing fidelity in the reads of the barcode, and long reads required for full coverage of the fragments. Furthermore, in many cases the fragment is required to sit multiple thousand bases away from the barcode in the viral vector. This is outside the usable range of most next-generation sequencing technologies. Here we have therefore validated two approaches for sequence truncation (library 1 and 2–4 respectively). The second sequence truncation method was here developed as a consequence of the imperfect results from the first (previously broadly utilised) approach. Sequence truncation aims to bring the genomic fragment next to the barcode so that they can be sequenced together in a short amplicon. However, it also has other benefits *e.g.,* removing vector derived terminal repeats (LTRs or ITRs) that may disrupt amplicon generation and/or sequencing. The basic principle is that each barcoded plasmid library is divided into two aliquots where the first one is utilized “as is” to perform the experiment (*e.g.,* used to produce a viral vector library for *in vivo* applications) with the readout of only the barcodes (from DNA or RNA). The second aliquot is prepared for sequencing to generate a lookup table for barcode and fragment combinations. It is to the latter where the sequence truncation is applied.

To assess the quality of the plasmid libraries and the successful sequence truncation, we have here implemented a novel quantitative measurement called the “purity parameter”. The purity parameter is a fraction defining the relative number of reads having the same consensus genomic sequence (see materials and Methods) out of the total reads for that specific barcode. This parameter ranges from 1 and towards 0 where 1 denotes a barcode where all reads have the same genomic sequence (±the accepted read alignment error). This is presented as a beanplot for each library where singlet reads (*i.e.,* barcodes represented only by a single read, most often the result of a sequencing error) have been excluded, as the purity function cannot be determined in those reads. When interpreting the beanplots, a library with all barcodes with a purity value of 1 indicates a perfect sequence truncation, flawless sequencing and a cloning methodology where each barcode is used only once.

**Figure 3:**
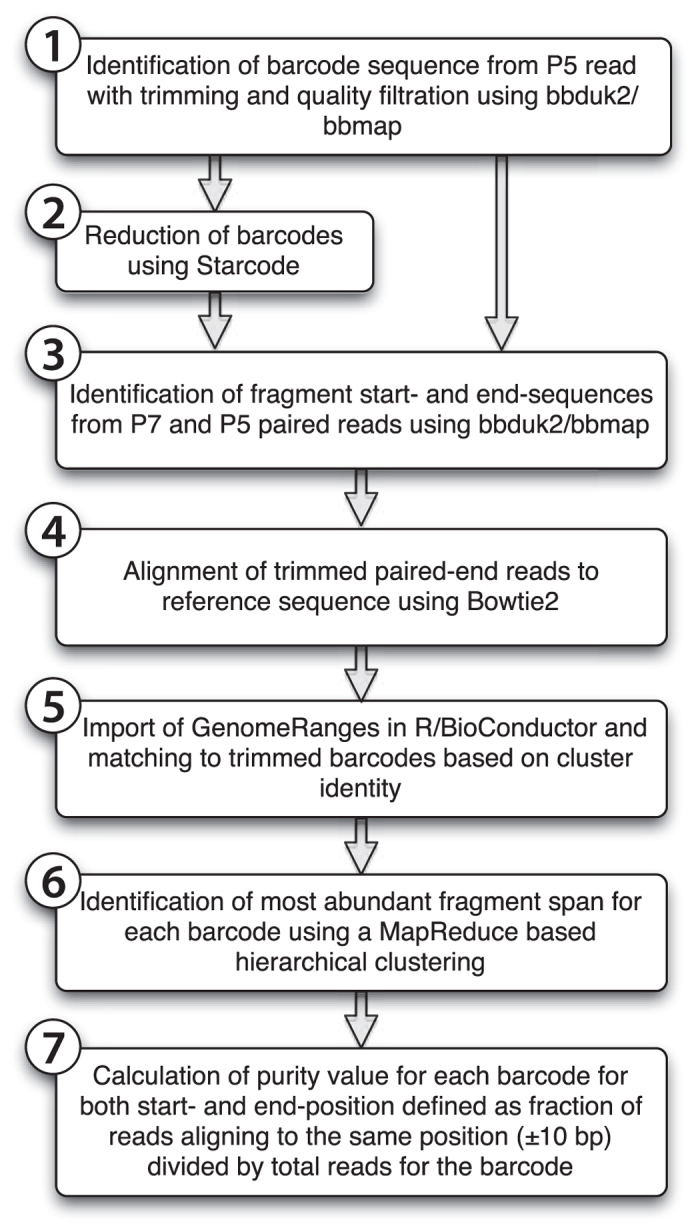
Barcoded plasmid library analysis workflow. The sequence analysis framework utilized here is implemented inside the R statistical package with Bioconductor ShortRead package using a MapReduce programming philosophy. However critical components were also implemented from stand-alone software projects such as bbmap^19^, Starcode^15^ and Bowtie2 (see Materials and Methods for details). In the current implementation it is optimized to run on a multi-core server with shared memory but the workflow is well suited for distributed MapReduce implementations as well. It contains two mapping steps and two reduction steps. **(4)** *Map 1;* Alignment of the each contained genomic fragment per paired read to a reference using Bowtie2 and translate into the representation of a GenomicRange object. **(5)** *Map 2;* replacement of cluster identity of each paired read with the contained genetic barcode. **(6)** *Reduce 1;* Sort on the barcode sequence and cluster GenomicRanges objects with the same barcode into discreet lists. **(7)** *Reduce 2;* Through hierarchical clustering determine the consensus GenomicRange per unique barcode and calculate a purity variable. With this structure, a complete analysis can be conducted on 30 million reads of a library with 3 million unique barcodes in less than 6 hours. Two thirds of this time is consumed by the *Reduce 2* calculation, which has an execution time linear to the number of unique barcodes and is less sensitive to sequencing depth.

The linkage of barcodes to genomic fragments and the consecutive calculation of the purity parameter was conducted inside the R statistical package^18^ (Fig. 3) with Bioconductor ShortRead package with critical components implemented from stand-alone software projects such as bbmap^19^, Starcode^15^ and Bowtie2^20^ (see Materials and Methods for details or the full Git repository at https://bitbucket.org/MNM-LU/workflowlibrary4).

### Barcode characterization

**Figure 4:**
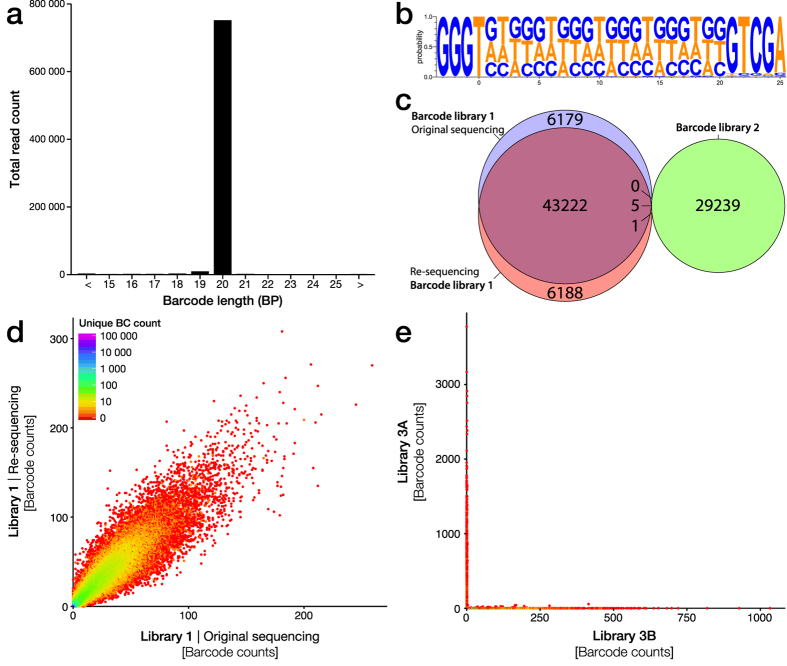
Characterization of molecular barcode diversity. **(a)** Length analysis of unfiltered sequencing data from the Ion Torrent platform from library 1 after trimming of the flanking sequences of the degenerate barcodes. This revealed that the vast majority of barcodes are synthesized to the specification of 20 bp. **(b)** As homopolymer sequences commonly result in sequencing errors in the form of insertion/deletion errors, a cycling nucleotide exclusion paradigm (IUPAC ambiguity code VHDBx5) was used during degenerate primer synthesis to avoid homopolymers longer than three nucleotides. This resulted in close to uniform distribution of the three nucleotides per position with a slight bias for the Guanine, visualized by Weblogo3.3 based on the first 40 000 unique barcodes in library 1. **(c)** Reproducibility in barcode identification was assessed through comparison of the original Ion Torrent sequencing of library 1 with the re-sequencing conducted using the MiSeq platform and compared those unique barcodes to those found in the library 2 sequenced using the Ion Torrent platform. **(d)** Analysis of the sequence/re-sequence correlation of the read count per unique barcode for library 1. **(e)** Using two larger libraries (library 3A and 3B using identical cloning but in two separate reactions) we found near complete orthogonality meaning that each unique barcode was only found in one or the other library. These libraries are estimated to contain around 45 000 and 43 500 unique clones each.

The barcode structure in this experiment was generated as described above through single cycle PCR extension using an oligonucleotide with 20 degenerate bases flanked by static regions (5′ domain complementary region and the AttB2 sequence). As they include a long degenerative sequence, the real length of each oligonucleotide cannot globally be confirmed. However, analysis of the unfiltered sequencing reads, covering the barcode region, revealed that the vast majority of barcodes are synthesized to the specification of 20 bp (Fig. 4a). Common sequencing errors on NGS technologies are insertion/deletion errors, being the most common error on the Ion Torrent/PacBio platforms and the second most common on the Illumina platform. These errors also become more common with homopolymer sequences. Therefore, we chose to design our barcode structure based on a base cycling paradigm, systematically excluding one of the four nucleotides in each oligonucleotide extension step. This barcode design, described as (VHDB)x5, still allows for a very high diversity (3^20^ ≈ 3.5 billion unique sequences) while guaranteeing that homopolymers longer than three nucleotides are never formed inside the barcode. Through Weblogo3.3 based visualization of unique barcodes in library 1, we found that this design is largely retained in the actual barcode sequencing but with an overrepresentation of G and T over C and A (Fig. 4b). Note that the read count per barcode is not taken into account in this logo and thus PCR or sequencing bias should have less effect on this skew.

Two key criteria for barcode-based applications are; reproducible quantification and complete orthogonality between libraries (*i.e.,* that the same barcode is not found in different libraries). To study these two factors, we first compared the original Ion Torrent sequencing of library 1 with the re-sequencing conducted using the MiSeq platform. Indeed, we found that the vast majority of barcodes in library 1 were identified using both the Ion Torrent and Illumina platform (Fig. 4c) with a reasonably good correlation in relative abundance (Fig. 4d). In a second step, we compared the unique barcodes in library 1 to those found in the library 2, also sequenced using the Ion Torrent platform. Encouragingly, only six barcodes were overlapping between library 1 and 2 out of the 84 834 unique barcodes assessed (Fig. 4c). Using two larger libraries (library 3 A and 3B using identical cloning but in two separate reactions) we found similar complete orthogonality and the few barcodes that were found to overlap displayed non-correlated relative abundance in the two libraries (Fig. 4e), indicating that they most likely appeared in the second library due to sequencing errors and are not in fact true barcodes. Thus, they can easily be filtered out from the non-relevant library post-hoc.

### Ligation of digested plasmids lead to a very high heterodimer formation

**Figure 5:**
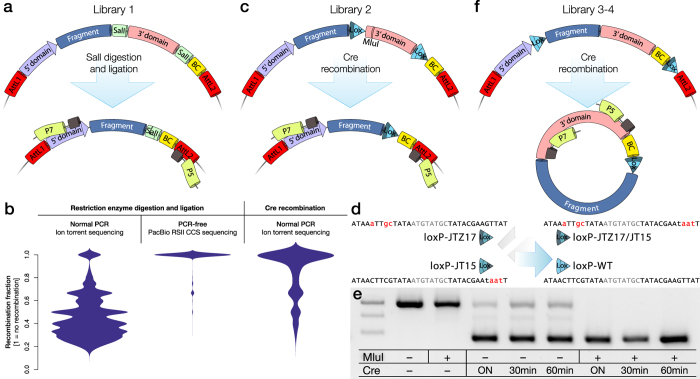
Design and validation of three alternative approaches for sequence truncation prior to library sequencing. **(a)** In library 1 we utilized a sticky-end restriction enzyme (SalI) digestion and T4 ligation to remove the static sequence separating the variable genomic fragment of interest with the degenerate DNA barcode (BC) sequence. **(b)** When the library plasmid was truncated, the barcode could be sequenced together with the variable genetic fragment to generate a look-up table (LUT) using the Ion Torrent sequencing platform. However, the sequencing results from library 1 displayed extensive recombination between barcode and fragment (left in B). This was confirmed to not have been originating from the cloning process through the use of PCR free sequencing using the PacBio sequencer on the non-digested plasmid (centre in B). Using the Cre-recombinase based approach in C, this recombination could be significantly reduced (right in B). **(c)** In library 2 we replaced the restriction enzyme approach with a Cre-recombinase approach where the same intervening static sequence is removed through the recombination between two loxP sites. **(d)** In the Cre-based designs we utilize a combination of two mutant loxP sites; loxP-JT15 and loxP-JTZ17 which promote superior Cre-induced recombination compared to wild-type loxP sites as the resulting double-mutant loxp-JT15/JTZ17 has lost the binding capacity of the Cre-recombinase making the recombination a unidirectional event. **(e)** The loxP-JT15/JTZ17 combination resulted in 79%, 81% and 89% recombined product with 30 minute, 60 minute and overnight Cre-recombination respectively. With restriction enzyme digestion (MluI, which cuts inside the 3′ domain) of the remaining un-recombined product, the remaining fraction of un-truncated plasmid could be removed (last three columns in E). The expected bands are 1007 bp and 464 bp respectively. **(f)** In the third and final design, we generated two libraries (3 & 4) where the design is reversed to that the fragment together with the barcode is excised into a mini-plasmid after Cre-recombinase exposure with the fragment and barcode in close proximity.

The most straightforward and broadly utilized sequence truncation method is the use of restriction enzyme digestion, gel extraction and T4-ligase re-circularization (Fig. 5a)^9^. Our library 1 was designed to utilize this approach and we assessed this library using Ion Torrent sequencing followed by the above described analysis framework. This sequencing technology generates a single direction read covering the barcode and gene fragment. Strikingly, the analysis of this library, as visualized using a bean plot of the purity parameter for each barcode, showed that next to none of the barcodes were retained as pure (left in Fig. 5b). The vast majority of barcodes pointed to multiple fragments with no internal logic. To determine if this was truly due to hetero-dimer formation in the ligation reaction or if this is a sequencing artefact of the Ion Torrent platform (where polyclonal beads are known to be generated in the preparation step) or if the approach of plasmid barcoding did not lead to unique utilization of each barcode, we performed a PCR-free CCS sequencing on the PacBio platform. The PCR free analysis of the undigested library 1 showed that indeed the plasmid library contained nearly only monoclonal barcodes (centre in Fig. 5b) *i.e.,* an average purity near one. Thus, the artefact observed in the Ion Torrent sequencing of library 1 must be due to either heterodimer formation in the RE-digestion and ligation protocol or the PCR preparation and sequencing. To exclude the sequencing technology, we sequenced the same library using the Illumina MiSeq platform but here the purity bean plot was near identical to that of the Ion Torrent (not shown). To confirm if the majority of error came from the restriction enzyme and ligation approach, we generate a second library (library 2) using the same genomic template for fragment generation, but in this case replaced the SalI digestion with the unidirectional Cre recombination^21^ (Fig. 5c).

Thompson *et al*.^22^, have performed a systematic analysis of loxP mutant forms and found a very advantageous combination; loxP-JT15 and loxP-JTZ17. When placed in line, this pair promotes far superior Cre-induced recombination compared to wild-type loxP sites. Furthermore, the resulting double-mutant loxp-JT15/JTZ17 has lost the binding capacity of the Cre recombinase making the recombination a unidirectional event and thus the equilibrium is pushed even further towards the truncated form (Fig. 5d). In this project we introduced the mutant loxP-sites into two different plasmid designs (Fig. 5c,f). In library 2, the loxP-JT15 was placed just 3′ of the fragment and the loxP-JTZ17 5′ of the barcode (Fig. 5c). With the addition of Cre-recombinase, the fragment and barcode were brought together in the original plasmid through excision of the interleaving 3′ domain by recombination. While the wild-type loxP recombination efficiency *in vitro* (using Cre recombinase on plasmid or linear DNA) is normally in the order of 20% efficiency^23^ the loxP-JT15/JTZ17 combination resulted in 79%, 81% and 89% recombined product with 30 minute, 60 minute and overnight Cre-recombination respectively (Fig. 5e). With the addition of a digestion of the remaining un-recombined product (using a restriction enzyme cutting inside the 3′ domain, in this case MluI) the remaining fraction of un-truncated plasmid could be removed (last three columns in Fig. 5e). After PCR preparation of this Cre-recombined plasmid library, we found a vastly improved purity bean plot of this library even using the Ion Torrent platform compared to library 1 (Fig. 5b). However, in some experimental settings, such loxP placement may not be ideal. Therefore, we also developed a third design (library 3–4) where the loxP-JTZ17 is placed 5′ of the fragment and the loxP-JT15 placed 3′ of the barcode (Fig. 5f). In this design, the fragment and barcode are brought together in the excised miniplasmid and not in the original plasmid and in this case also in a front-to-back orientation.

### Development of a simple and scalable emulsion PCR protocol for library sequencing

Replacement of the digestion and ligation approach with the Cre-recombinase system for amplicon truncation greatly improved the purity in short read based NGS technologies that depend on PCR for sample preparation. However, this still cannot match the purity of PacBio sequencing and we speculated that this may be a result of template switching in the PCR preparation of the library samples.

**Figure 6:**
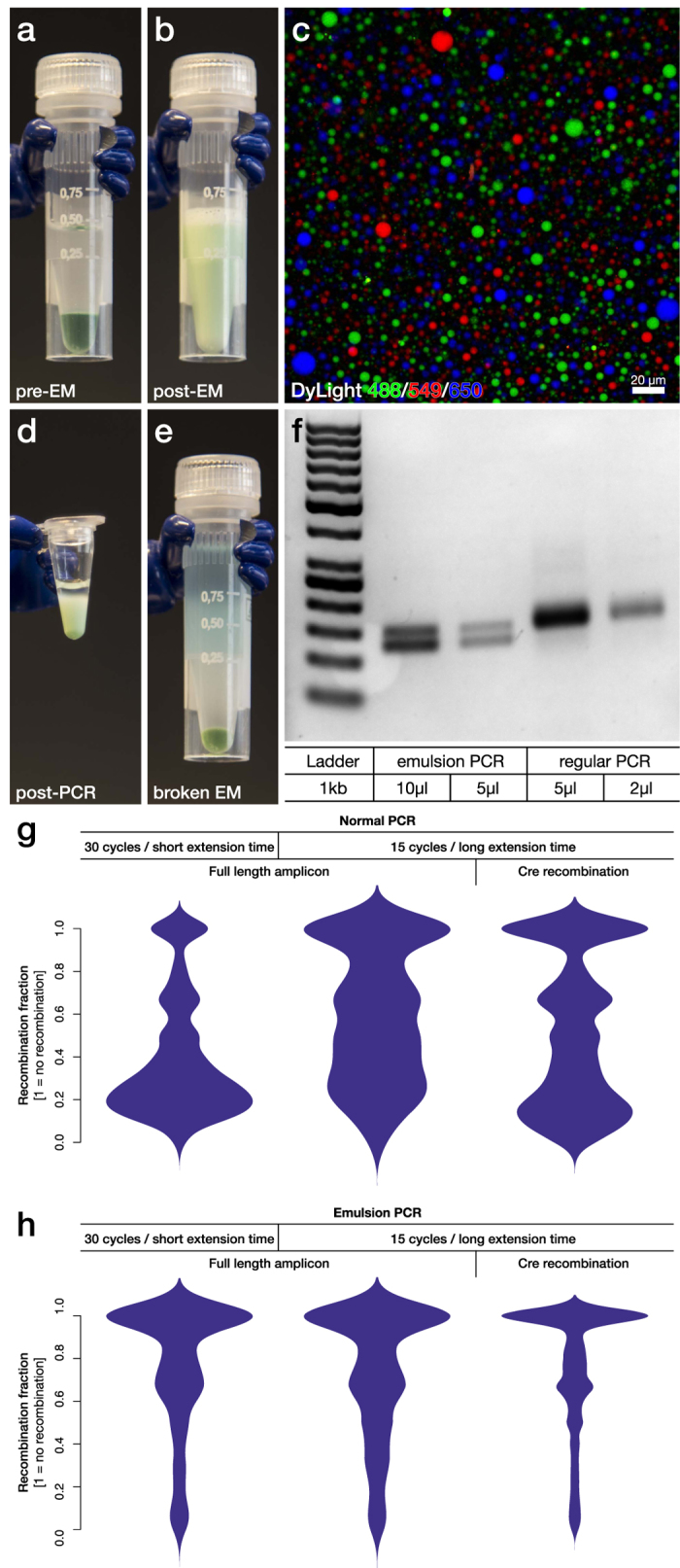
Development and characterization of an optimized emulsion PCR protocol. **(a)** An optimized, large scale, emulsion PCR protocol was developed to reduce template switching in the generation of amplicons for Ion Torrent and Illumina sequencing. A 50 μl PCR reaction (Phusion Hot Start with green buffer) was mixed with mineral oil and surfactant. **(b)** The mix was converted into a large scale emulsion using a Fast Prep homogenizer in 5 minutes. **(c)** To evaluate the stability and size distribution of the formed micelles, three separate PCR reactions were prepared and labels with different water soluble fluorophores (DyLight 488, 549 and 650 respectively) and made into three emulsion reactions. After mixing together the three emulsions through repeated pipetting the reaction was imaged and quantified using laser-scanning confocal microscopy emulsion. Quantification resulted in a mean diameter of the micelles of 3.7 ± 2.3 μm and a total micelle count of 2.6 × 10^8^ per individual reaction. **(d)** The emulsion was then divided into 6 individual PCR tubes and covered with mineral oil, which remain stable after PCR. **(e)** Isobutanol was then used to break the emulsion. **(f)** The compartmentalization of a PCR reaction by emPCR enables even amplification of an equimolar mixture of two oligonucleotides (126 and 150 bp long) with identical flanking sequences but with known difference in PCR efficiency. **(g)** Formation of chimeras due to template switching was analysed through a comparative experiment utilizing the long amplicon library 3, containing a 1 kb long stretch of constitutive backbone which can be removed using Cre-recombinase (Fig. 3c). With regular PCR, this library displayed extensive template switching regardless of PCR protocol or length of constitutive sequence. **(h)** Using emPCR on the other hand, the template switching could be significantly reduced to a level where it became insensitive to both changes in PCR protocol and constitutive sequence length.

Towards this goal we set out to develop a large scale emulsion PCR protocol that would allow the division of the sample into >10 million discreet reactions while retaining the PCR efficiency. This protocol is an optimized version of two previously published protocols^24^,^25^. This allows for a 50 μl PCR reaction (using Phusion Hot Start with a green buffer) combined with mineral oil and cheap surfactant (ABIL WE 09) (Fig. 6a) to be converted into a large scale emulsion using a Fast Prep homogenizer in only 5 minutes (Fig. 6c). To optimize the size distribution of the individual reactions (micelles) in the emulsion, three separate PCR reactions were prepared and labelled with different water soluble fluorophores (DyLight 488, 549 and 650 respectively). This allowed for the evaluation of emulsion stability and accurate estimation of reaction volumes using laser-scanning confocal microscopy. The three emulsion reactions were prepared independently and then mixed together by repeated pipetting. Using this optimized protocol, the emulsion displayed excellent stability with no micelle fusion (*i.e.,* no fluorophore overlap) and a mean diameter of the micelles of 3.7 ± 2.3 μm (Fig. 6c). This corresponds to a reaction volume of 20 femtolitre (2 × 10^−14^ litre) and a total micelle count of 260 million individual reactions from a 50 μl initial PCR reaction. The emulsion was then divided into 6 individual PCR tubes and covered with mineral oil. Even after the longest PCR protocol utilized in this study, the emulsion displayed excellent stability (Fig. 6d) and the complete aqueous phase could easily be recovered using isobutanol to break the emulsion (Fig. 6e). To visualize the efficiency of emPCR utilizing the novel protocol, we performed comparative PCR reaction on a equimolar mixture of two oligonucleotides (126 bp and 150 bp long respectively) with identical flanking sequences but with known difference is PCR efficiency. We found that the amplification rate of the emPCR was around 50% of the regular PCR, but that the amplification bias in the regular PCR was completely abolished using the emulsion as there is no competitive bias in each micelle (Fig. 6f).

Previous studies have reported amplicon length, number of amplification cycles and extension time as being three key determining parameters for the formation of chimeras due to template switching^12^,^14^,^26^,^27^. Thus, we performed a comparative experiment utilizing our library 3, *i.e.,* a long amplicon library where the long stretch of constitutive backbone, separating the genomic fragment and the barcode would be excised using Cre-recombinase (Fig. 5f). With this library we could compare long constitutive sequence amplification (700 bp) to short (40 bp) using the same variable sequences and the same exact library. For the long amplicon we also varied the number of amplification cycles (15 and 30) and finally the extension time varying between 1 min/kb to 8 minutes/kb. Three different combinations were evaluated under normal PCR and the new emPCR protocol. These were amplified from the same plasmid prep in parallel, indexed with Nextera primers (all using emPCR and 5 cycles) and sequenced in the Illumina NextSeq using paired-end sequencing. Using normal PCR, it is evident that PCR recombination occurs to a significant degree under every setting (Fig. 6g), but that an increase in extension time and reduction in amplification cycles can reduce the magnitude of the recombination. Using emPCR on the other hand, the recombination events are far less frequent (Fig. 6h) and the amplification is much less sensitive to PCR parameters. Truncation of the constitutive region using the Cre recombinase still however provides an advantage (right in Fig. 6h).

### Implementation and evaluation of an optimized library preparation protocol

**Figure 7:**
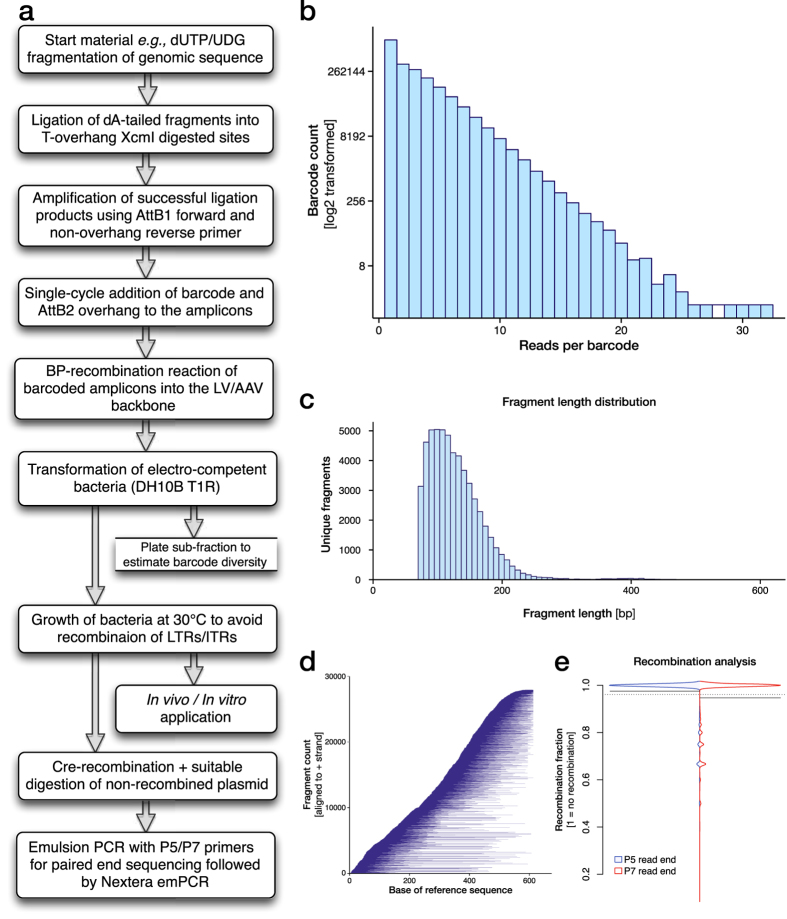
An optimized library preparation protocol. **(a)** An optimized workflow for the generation and characterization of plasmid libraries for expression in viral vectors has been generated based on the observations in this paper. **(b)** As proof-of-principle data, we generated an AAV plasmid library, (library 4) applying all optimized steps and characterized it by NextSeq paired-end sequencing. This experiment yielded 2.5 × 10^6^ ampicillin resistant clones per electro-competent bacteria transformation with barcode counts that closely follow a Poisson distribution of with a small inflation of singlet reads and **(c)** a smooth distribution of fragment lengths cantered around 100 bp. **(d)** This method showed very low sequence bias with the entire reference sequence evenly covered by the fragments in the barcoded library with 27928 unique fragments oriented in the cis (+) and 27997 in the trans (−) orientation. **(e)** With this optimized protocol we achieved a very high purity of all fragment alignments per unique barcode, now at the same level as the PacBio PCR free sequencing but using a much cheaper paired-end sequencing (compare centre in Fig. 5b with e).

Based on the observations in this paper we now propose an optimized protocol for the generation and characterization of plasmid libraries (Fig. 7a). As proof-of-principle, we generated an AAV plasmid library (library 4), applying all optimized steps and characterized it by NextSeq paired-end sequencing.

This library process can be performed in a standard molecular biology laboratory from start to finish in less than two weeks and has in our experiments yielded 2.5 million ampicillin resistant clones per electro-competent bacteria transformation. Thus, it is feasible to scale this to 10 million and beyond if required.

The resulting libraries closely follow a Poisson distribution of barcode counts with a small inflation of singlet reads (Fig. 7b) and a smooth distribution of fragment lengths centred around 100 bp (Fig. 7c). Purification of fragments was conducted before dA/dT ligation to remove fragments smaller than 50 bp (using AMPure beads). This method of fragmentation and ligation showed very low sequence bias with the entire reference sequence evenly covered by the fragments in the barcoded library (Fig. 7d). For this library, covering a 600 bp reference sequence, this resulted in 27928 unique fragments oriented in the cis (+) orientation and 27997 in the trans (−) orientation, showing that the ligation process does not induce bias in insertion orientation.

With this protocol, we achieve a very high purity of all fragment alignments per unique barcode (Fig. 7e), now at the same level as the PacBio sequencing, but utilizing Cre-recombinase based truncation and much cheaper paired-end sequencing (compare centre in Fig. 5b with Fig. 7e). However, one challenge with oversampling of sequencing depth is how to determine the true library size based on the unique barcode count. With the increased sequencing depth of the NextSeq over *e.g.,* the MiSeq comes also a reduction in sequencing fidelity. This is observed through evaluation of unique barcodes found at different phred filtration levels. Without filtration for phred quality inside the identified barcode region, 9 762 153 unique barcodes were found in the 30 million NextSeq reads for this library. This estimation is highly improbable based on the 2.5 million clone count observed during the library preparation. With an increase in phred filtration level, the unique barcode count is reduced to 7 173 750 (phred 20) and 4 542 009 (phred 30). However, even at this high level of read quality filtration, the unique barcode count is still higher that the physical measurement.

A number of strategies have been proposed for sequence clustering with the aim to reduce *de novo* sequences in NGS data sets induced by sequencing errors^28^. One algorithm in particular “Starcode” has been designed to tackle the challenge of clustering degenerate sequences such as barcodes without any possibility of reference alignment^15^. However, no conclusive evidence exists on the validity of the outcome from this algorithm, *i.e.,* what fraction of “false clustering” does this induce at different clustering stringency levels (determined by the accepted Levenshtein distance between barcodes considered to belong the same cluster). In this library, we here have a unique possibility to conclusively characterize this component as the sequencing data contains two linked sets of hypervariable regions; the fragment originating from the reference sequence and the degenerate barcode region with a totally unknown sequence but which is linked to the fragment.

The Starcode software package incorporated two sequence clustering algorithms. For multi-purpose clustering problems, Starcode implements an algorithm they call “sphere clustering” where sequences are sorted by frequency of occurrence and then through descending frequency occurrence claims all its similarity matches (*i.e.,* all matches form a cluster of defined radius based on the Levenshtein distance). Claimed sequences can belong to only one cluster. The more advanced algorithm in Starcode, tailored for barcode clustering, is based on a variation of the Needleman–Wunsch (NW) algorithm and provides an message passing clustering methodology determining the Levenshtein distance in every pair comparison. While computationally challenging, this approach should allow for a clustering of higher validity, but is to date not shown in practice.

Here we compared the sphere clustering to the message passing clustering in Starcode and utilized the purity parameter as a readout of true, versus false positive clustering achieved by the algorithms *i.e.,* if two truly unique barcodes are falsely clustered together, this would reduce the purity measurement as they would point to different fragments (both alignment and orientation is considered so the library contains 55 925 unique fragments).

**Figure 8:**
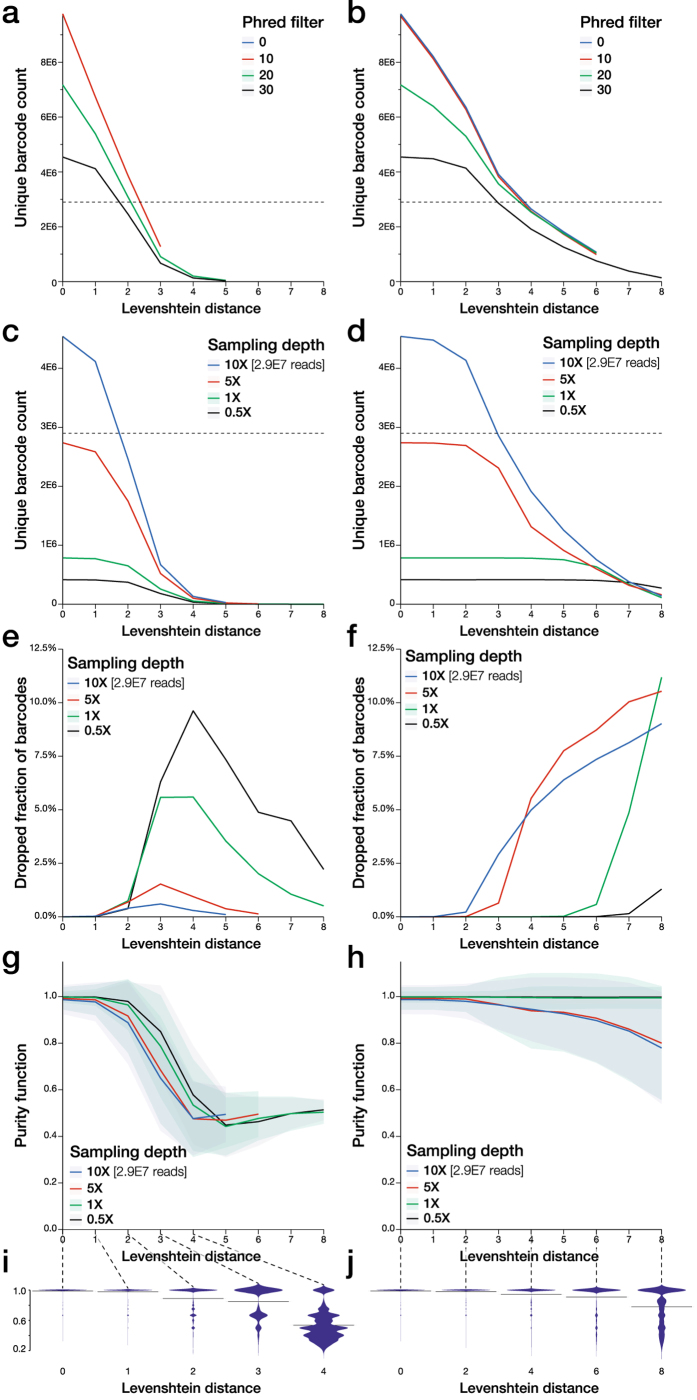
Validation of the Starcode message passing clustering algorithm using library 4. **(a)** To determine the true identity of all unique barcodes and the real library content, we evaluated two clustering algorithms on the NextSeq sequencing data set from library 4. The NextSeq identified 9 762 153 unique barcodes although only 3 × 10^6^ clones were observed during the library preparation. The Starcode software package multi-purpose “sphere clustering” reduced the number of unique barcode sequences down to the expected number (dashed line in a–d) at a Levenshtein distance around 2. **(b)** The more advanced algorithm in Starcode, tailored for barcode clustering based on a variation of the Needleman–Wunsch (NW) algorithm and provides an message passing clustering. This required a Levenshtein distance of around 4 to reach the expected barcode count. Due to the non-linear increase in memory requirements with the increase in Levenshtein distance threshold of these algorithms, the calculations failed to complete at some of the highest levels with the 196 gb ram and 1 Tb SSD ram swap file we had at hand for this computation. **(c,d)** Both algorithms require a read depth of 10x the clone count to recover all unique barcodes as seen through subsampling of the read counts (evaluated after filtration by phred of min 30). **(e,f)** Both algorithms include an evaluation of ties and discards barcodes that are ambiguous at the point of clustering, *i.e.,* at equal distance to two generated clusters. However, the sphere clustering displays an inverted U shape of the fraction of discarded barcodes while compared to the saturating rate in the message passing clustering algorithm. **(g,i)** However, the biggest difference between the two algorithms was observed by studying the purity function. If two, truly unique, barcodes are falsely clustered together, this would reduce the purity measurement as they would point to different fragments. While an observable reduction in barcode purity was seen already at a Levenshtein distance threshold of 2 and completely breaks down at a distance of 4, **(h,k)** the message passing clustering algorithm manages to retain the barcodes highly pure up to a distance of 6.

At a Levenshtein distance between 2 and 4, both algorithm reduced the number of unique barcode sequences down to the expected number (based on the physical count) regardless of the a priori phred filtration (Fig. 8a,b). To study the effect of sequencing depth on the sequence reduction capability of the algorithms (higher depth for the same true library diversity should make the clustering of true barcodes easier), we then simulated different sampling rates. As expected, the clustering was more substantial at higher sequencing depth and was more promiscuous using the sphere clustering algorithm already at a low Levenshtein distance threshold (Fig. 8c,d). Both algorithms include an evaluation of ties and discards barcodes that are ambiguous at the point of clustering, *i.e.,* at equal distance to two generated clusters. However, the sphere clustering displays an inverted U shape of the fraction of discarded barcodes compared to the saturating rate in the message passing algorithm (Fig. 8e,f). However, the biggest difference between the two algorithms was observed by studying the purity function. While an observable reduction in barcode purity was seen already at a Levenshtein distance threshold of 2 and completely breaks down at a distance of 4 (Fig. 8g,i), the message passing clustering algorithm manages to retain the barcodes highly pure up to a distance of 6 (Fig. 8h,k). At distance 3–4 this algorithm presents an optimum, where the number of clustered unique barcodes closely match the physical cluster count and purity is well maintained throughout the different sequencing depths and does not require a priori phred filtering of the reads. These data show that the message passing algorithm, used restrictively, is well suited for sequence clustering when studying barcode diversity and can significantly improve the reliability in the absolute counts.

## Discussion

In this paper, we have for the first time made a systematic characterization of methods for barcoded plasmid library generation and have for every step along the way evaluated the outcome with regards to library diversity. One often overlooked challenge is how to generate a true representation of the library (*i.e.,* the relationship between the referenced barcode and the exact functional sequence inserted), using labour- and cost-effective solutions. For low cost per sequence (more important than cost per base in library analysis studies), the Illumina sequence-by-synthesis (SBS) technology is currently without serious competition^29^. However, with this technology, preparation of sequencing material from both *in vitro* and *in vivo* samples require PCR amplification. As we have shown in this study, regular, single reaction PCR can induce serious errors in the representation of the library even when minimizing template concentration and amplification cycles. While we are far from the first to show this effect^12^,^14^,^26^,^27^,^30^,^31^, there has not been any thorough studies on how this affects the characterization of barcoded libraries and no alternative strategies have been described to fully mitigate this issue.

The sequencing using ultra-long reads with a PCR free single molecule sequencing like the PacBio CCS technology is here shown to be a very clean way to characterize libraries. Unfortunately, this technology, while providing unique value for certain applications, is still very expensive per read. A high diversity library with 2.5 million unique barcodes, sequenced at a depth of 10X (the absolute minimum to ensure read of most barcodes in the library), requires over 400 individual SMRT cells and would run into prohibitive cost (multiple of hundreds of thousand USD) and sequencing time. On the Illumina SBS platforms *e.g.,* the NextSeq utilized here, the same sequencing costs a few hundred USD and can be completed over night. With the recent announcement of the MiniSeq, low cost “personal sequencer” this may soon prove to be the optimal platform for plasmid library characterization and provide a very low cost entry point.

Readout of barcodes from *in vivo* samples, especially in applications of cellular fate mapping^32^,^33^,^34^ requires error free clustering. To date a number of approaches have been utilized to cluster barcodes, but all attempts have been applied on samples with unknown diversity. In this study we utilize the two-factor nature of the high diversity, barcoded fragment libraries to validate the approach of message passing clustering using the newly presented Starcode software. These data conclusively show that the message passing clustering algorithm, while not flawless, is sufficiently robust for degenerate barcodes even from relatively low quality sequencing data. However, these data also show that sequence clustering can induce false clustering if the Levenshtein distance threshold is increased. The optimal level is most likely dependent on the library diversity, sequencing depth and the length of the degenerate barcode. Thus, our strong recommendation is to utilize the here proposed method of clustering validation from a paired-end sequencing generated from the plasmid library *in vitro* and then cluster the *in vivo* sample data to this subset of “valid barcodes”. This removes uncertainty in the amount of false clustering.

Taken together, the optimized method we present here allows any laboratory to produce and sequence high diversity libraries using broadly available PCR based sequencing technologies. Especially the novel approach to emPCR ensures that this can be conducted cleanly and with low additional overhead with regards to both reagent costs and labour and the Starcode based barcode clustering can allow for clean and efficient assessment of the biological function.

## Materials and Methods

### DNA isolation and intron fragmentation

For library 1–3, genomic DNA was isolated from tail biopsies from mice (library 1–2) or rat (library 3). The isolated DNA was then used in a PCR reaction to amplify the required sequences using specific primers for the corresponding libraries. Library 4 was PCR amplified from plasmid DNA.

PCR product from each initial round of amplification was subjected to a second PCR, this time with varying dUTP concentrations, ranging from 0% to 100%. For this application Phusion U (Thermo Scientific) was used according to the supplier’s recommendations. The PCR products with dUTPs incorporated were then fragmented by adding Uracil-DNA-Glycosylase (UDG) (Sigma-Aldrich) followed by a 5 M NaOH treatment^16^,^35^. The DNA fragments were purified in Gene Jet spin columns (Thermo Scientific). Size distribution and fragmentation efficiency was validated by gel electrophoresis.

All experimental procedures performed in this study were conducted in accordance with relevant guidelines and regulations. The use of animal samples was approved by the Ethical Committee for the use of Laboratory Animals in the Lund-Malmö region.

### dA/dT ligation into zero-background vector

The zero-background cloning vector was digested with XcmI (NEB) restriction enzyme prior to the ligation. XcmI digestion separates the ccdB gene from the backbone and leaves T-overhangs on the backbone suitable for dA/dT ligation^17^. Digestion products were separated by gel electrophoresis and cleaved backbone without ccdB sequence was purified from gel.

Fragmented DNA (from dUTP PCR) was end-repaired and dA-tailed using NEBNext end-repair and dA-tailing module (NEB) following manufacturer’s protocols. Digested vector and dA-tailed fragments were ligated using T4 DNA Ligase (NEB) in a 1:6 (vector:insert) ratio at 16 °C over-night.

Library 1–2; ligation product was transformed into homemade chemically competent cells using 30 seconds heat shock at 42 °C followed by 1 hour incubation at 37 °C and then plated on agar plates. The following day, all colonies were scraped off the plates and prepped according to manufacturer’s protocol.

Library 3–4; no transformation after ligation was carried out, ligation product was instead used directly in the subsequent step.

### Barcode design

Barcodes were ordered as High Purity Salt Free purified oligos (Eurofins genomics) where the barcode length was 20 nucleotides and defined as ambiguity nucleotides by using the sequence V-H-D-B (IUPAC ambiguity code) repeated five times and flanked by static sequences containing 3′domain binding and AttB2 sequences.

### Addition of Barcodes and Gateway AttB-sites to fragment libraries

Library 1, Multi-cycle PCR – AttB1 and barcode + AttB2 were added in one PCR reaction using a forward primer containing AttB1-site and a reverse primer containing barcode followed by AttB2-site. The forward primer recognizes the 5′ end of the CMV promoter and the reverse primer the sequence downstream of the 3′ domain in the zero-background cloning plasmid.

Library 2–4, One-cycle Barcode PCR – In the first PCR, a forward primer containing AttB1 was used together with a reverse primer only complementary to the 3′ domain (i.e., no barcode/AttB2 overhang). This PCR was carried out by the Phusion U polymerase (Thermo scientific). The second PCR consisted of only a reverse primer extended to now contain a barcode and the AttB2-site, hence the name One-cycle Barcode PCR. Subsequently, a validation PCR was carried out using two primers complementary to the product produced after the two PCR’s to verify that both AttB-sites had been added.

### Gateway cloning into Lentiviral or Adeno Associated Viral vectors

Both LV-vectors (library 1 & 2) and AAV-vector (library 3) plasmids were designed to contain AttP1- & AttP2-sites, necessary for gateway cloning, flanking a ccdB negative selection gene.

The cloning was performed through a recombination reaction using Gateway BP Clonase II Enzyme Mix (Life Technologies). 150 ng of vector was used with 100 ng of the barcoded PCR-product from the previous step, according to manufacturer’s protocol. BP reaction was then used to transform Top10 competent cells (library 1–3) or electro-competent cells (library 4). A fraction of the transformation was plated on agar plates to estimate the number of cells containing the insert. The remaining larger fraction of the transformation was grown in 150 ml LB O/N at 32 degrees. After DNA isolation (prep) a validation PCR was carried out to verify the size of the inserted fragments in the libraries.

### Preparation of fragment libraries for sequencing

#### Sequencing using Ion Torrent

Plasmids from library 1 & 2 were digested using the SalI enzyme and the digestion products were separated by gel electrophoresis and cleaved backbone without 3′domain sequence was purified from gel. 75 ng of the linear product containing the fragment and barcode was circularized using T4 DNA Ligase (NEB) 16 °C over-night. The ligation product was treated with Lambda Exonuclease (NEB) and RecJF (NEB) for 16 hours at 37 °C^36^. The remaining, circularized product was PCR amplified with primers containing Ion Torrent sites P1 & A. The concentration was determined by Bioanalyzer and sequenced on Ion Torrent using a 316 V2 chip (Thermo Fisher). Sequencing was conducted as 500 bp long reads using an extended number of reagent flows in the 400 bp kit.

#### PCR free sequencing using PacBio RSII

Library 1 – Plasmids from the library were digested by unique restriction enzyme to result in products with the length averaging around 800 bp (min 696 bp, max 1726 bp). The fragments were then end-repaired using NEBNext end-repair kit (NEB). SMRTbell adaptor sequences (Pacific Biosciences) were then ligated on to the sequences according to the supplier’s protocol and sequenced using two SMRT-cells in the PacBio RSII sequencer.

#### Illumina sequencing

For library 2–4 a loxP-JTZ17-site was inserted between the 5′domain and the XcmI-ccdB-XcmI cassette of the cloning vector. The loxP-JTZ17 was used together with a loxP-JT15 site for recombination before sequencing of the libraries^22^. 750 ng DNA was incubated with 3 U Cre-recombinase (NEB), 10X buffer and water and incubated at 37 °C for 90 minutes. The reaction was terminated at 70 °C for 10 minutes. Non-recombined plasmid was linearized by ClaI or SmaI digestion followed by purification using DNA Clean & Concentrator (Zymo Research). Recombination efficiency was determined by gel electrophoresis. Subsequently, two PCR reactions were performed to add Illumina compatible ends to the fragments. A P5/P7 Illumina adapter PCR was performed followed by a Nextera XT index PCR where each library was labelled with a unique Nextera index. The library was then sequenced on an Illumina MiSeq (libraries 1–3) or NextSeq (library 4) sequencer.

### Emulsion PCR

The selected library was amplified by emulsion PCR in order to prevent template switching. The oil phase, made fresh and chilled on ice before addition to the aqueous phase, was composed of 92.95% Mineral Oil, 7% ABIL WE-09 and 0.05% Triton X-100, then thoroughly vortexed. The PCR mixture, or aqueous phase, was prepared in a 1.5 ml screw cap tube as follows: 0.5 μg/μl BSA, 0.2 mM dNTPs mix, 0.4 μM of each primer (P5/P7 PCR) or 5 μl of each primer (Nextera PCR), 0.5 μl Phusion HS II DNA polymerase (Thermo Scientific, Waltham, USA) and 1X Phusion Green buffer, together with either 1.5 × 10^9^ or 7.5 × 10^9^ plasmid copies (depending on the number of PCR cycles). dH_2_O was added to a final volume of 50 ul.

The water-in-oil emulsion was created by addition of 9 volumes of the oil phase on top of the aqueous phase and vigorous shaking of the tube in an MP FastPrep-24 Tissue and Cell Homogenizer (Hyland Scientific), for 5 min at a speed of 4 m/s. After shaking, the emulsified product was divided into six PCR tubes (50 μl to each) that were overlaid with 10 μl of mineral oil each to prevent the emulsion from breaking during the PCR program, due to extreme heating. Non-emulsified control reactions were utilized as controls.

#### Sequencing adapter PCR

In a first step, primers with Illumina P5 and P7 sequences were used to amplify the fragment and barcode from the plasmid. All reactions were prepared as duplicates. Two parallel different PCR programs were performed. The first PCR program was run for 30 cycles with the following cycling conditions: 95 °C for 2 min, 95 °C for 30 sec, 63 °C for 30 sec, 72 °C for 330 sec and 72 °C for 10 min. A ramp rate of 1 °C/s was applied from step 1 to 4. The second PCR program was run O/N for 15 cycles with the same cycling condition except for the extension step which corresponded to 72 °C for 20 min instead.

#### Breakage of the emulsion

Once the PCR programs were completed, the emulsion was broken. 100 μl of isobutanol (Sigma-Aldrich) was added to each PCR tube, pipetting up and down, and all PCR reactions, from the same initial PCR mix and program, were pooled together into a new 1.5 ml tube. 60 μl PCR buffer was added to increase the aqueous phase volume and facilitate its recovery. The sample was then mixed thoroughly by vortexing for 1 min and centrifuged at 16,000 × g for 2 min. The resulting upper- and interphase, were discarded, keeping the bottom phase, containing the PCR mix.

#### Purification of PCR samples

Agencourt^®^ AMPure XP purification kit (Beckman Coulter, Brea, USA) was used for purification of the samples as indicated by the manufacturer. The resulting product was re-purified through DNA Clean & Concentrator (Zymo Research) commercial kit. 1% agarose gel electrophoresis was performed for validation of PCR products.

### Nextera PCR

The purified samples were used as template for a second emulsion PCR round in which the Nextera Index primers were used for amplification. The same procedure described above for the P5/P7 PCR was followed but using 2 × 10^10^ amplicon copies per emPCR reaction. The PCR program run O/N for 15 cycles with the following cycling conditions: 95 °C for 2 min, 95 °C for 30 sec, 65 °C for 30 sec, 72 °C for 20 min and 72 °C for 10 min. A ramp rate of 1 °C/s was applied from step 1 to 4. The samples were purified using SPRIselect reagent kit (Beckman Coulter) as described by the manufacturer.

### Data assessment workflow

A complete interaction free workflow was implemented using the R statistical package together with a number of packages from the Bioconductor repository. From these scripts, a number of broad–utility external applications (bbmap, Starcode and bowtie2 and samtools) were called and output returned to R for further analysis. This is publically available as a Git repository at https://bitbucket.org/MNM-LU/workflowlibrary4.

In brief; barcode and sequence identification, trimming and quality filtration was conducted using the bbmap software package^19^, which allows for kmere matching of known backbone sequences against the paired reads. The column based synthesis process of 20 nt barcode primer utilized in this study does not allow for exclusion of primers not containing an exact 20 bp barcode. Thus in the initial step we identified the real world length distribution studying the remaining sequence after trimming of flanking sequences around the barcode. The vast majority of barcode reads were sequenced to the length of 20 with most barcodes of a deviating length ending up being 19 bp long (Fig. 5a). Thus, for all analysis in this study, length filtration of 18 ≤ BC ≤ 22 was applied.

The genomic sequence fragments were similarly isolated using the bbmap software package, but this time without any application of length restrictions.

The key component of the R-based analysis framework is a parallelized implementation of the MapReduce programming philosophy^37^,^38^. In this case the workflow has two mapping steps and two reduction steps:

*Map 1;* Trimmed fragment sequences were piped to Bowtie2 for paired-end or single-read alignment (depending on the sequencing technology utilized) against the genomic reference sequence. The SAM file output was filtered for unique valid pair alignment only and sorted into a BAM file using samtools and read back into R as GenomicRanges. *Map 2;* The sequencing cluster identity information was then utilized to back-project each extracted barcode to the corresponding GenomicRange.

*Reduce 1;* the GenomicRanges were split into discreet lists each containing one unique barcode and lists of length = 1 (*i.e.,* containing a single read) were extracted. *Reduce 2;* the remaining lists were analysed in parallel to perform a hierarchical clustering of GenomicRanges, counting and ordering identical GenomicRanges. The most abundant range defined the consensus Genomic read for each barcode. In addition, range similarity was analysed using fragment start/end position of ±20 bp as an inclusion criterion. The count of all GenomicRanges not being identical to the top consensus range, but fulfilling the inclusion criteria were added to the consensus read count. In addition, the count of all reads in the list was added to the consensus GenomicRange to provide a basis for calculation of the quantitative “Purity” parameter (see below).

In all, this framework allows for the reduction of sequencing data from thee key/value pair of: NGS cluster identity/read to Unique barcode/consensus GenomicRange with a purity assessment of fidelity.

### Data Availability

The datasets supporting the conclusions of this article are available in the NCBI Sequence Read Archive (SRA) with the Accession numbers: PRJNA320207 (complete BioProject), SAMN04924907 and SAMN04924908 (library 1), SAMN04924909 (library 2), SAMN04924910, SAMN04924911, SAMN04924912, SAMN04924913, SAMN04924914 and SAMN04924915 (library 3) and SAMN04924916 (library 4). The R-based workflow is publically available as a Git repository at https://bitbucket.org/MNM-LU/workflowlibrary4.

## Additional Information

**How to cite this article**: Davidsson, M. *et al*. A novel process of viral vector barcoding and library preparation enables high-diversity library generation and recombination-free paired-end sequencing. *Sci. Rep.* **6**, 37563; doi: 10.1038/srep37563 (2016).

**Publisher’s note:** Springer Nature remains neutral with regard to jurisdictional claims in published maps and institutional affiliations.

## Figures and Tables

**Table 1:** Descriptive summary of the utilized protocol for each of the described plasmid libraries.

Library	Vector	AttB-PCR	Bacteria	CFU/ug	# Colonies	# BP	# TF	Col/BP	Col/TF
Library 1	Fig. 5a, LV SalI	Multi-cycle PCR	Chem. comp. (TOP10™)	1E8*	34640	2	20	17500	1750
Library 2	Fig. 5c, LV Cre	One-cycle PCR			26500	6	42	4500	650
Library 3	Fig. 5f, AAV Cre			1E9	45000	1	5	45000	9000
Library 4			Electro-comp. (DH10B T1R)	3E10	2952000	2	1.2	1476000	2460000

^*^Approx. of lab generated competent cells. #BP refers to total number of BP reactions and #TF refers to total number of transfections.

**Table 2:** Utilized strategies for insertion of genomic sequences into cloning plasmids.

Library	Fragment	Backbone preparation		Insertion method	Vector	Col/TF
Library 1	Adapter ligation	Linearization	Restriction enzyme	In Fusion	LV	25
	A-tailing	T-tailing	Terminal Transferase	dA/dT-ligation		0
					Cloning	20
			Taq polymerase		LV	50
					Cloning	100
			XcmI			>1000
